# Predicting Plant Diversity Patterns in Madagascar: Understanding the Effects of Climate and Land Cover Change in a Biodiversity Hotspot

**DOI:** 10.1371/journal.pone.0122721

**Published:** 2015-04-09

**Authors:** Kerry A. Brown, Katherine E. Parks, Colin A. Bethell, Steig E. Johnson, Mark Mulligan

**Affiliations:** 1 School of Geography, Geology and the Environment, Centre for Earth and Environmental Science Research (CEESR), Kingston University, London, United Kingdom; 2 Centre for Environmental Sciences, Faculty of Engineering and the Environment, University of Southampton, Southampton, United Kingdom; 3 High Performance Computer Cluster, Faculty of Science, Engineering and Computing, Kingston University, London, United Kingdom; 4 Department of Anthropology and Archaeology, University of Calgary, Calgary, Alberta, Canada; 5 Kings College London, Department of Geography, London, United Kingdom; University of New England, AUSTRALIA

## Abstract

Climate and land cover change are driving a major reorganization of terrestrial biotic communities in tropical ecosystems. In an effort to understand how biodiversity patterns in the tropics will respond to individual and combined effects of these two drivers of environmental change, we use species distribution models (SDMs) calibrated for recent climate and land cover variables and projected to future scenarios to predict changes in diversity patterns in Madagascar. We collected occurrence records for 828 plant genera and 2186 plant species. We developed three scenarios, (i.e., climate only, land cover only and combined climate-land cover) based on recent and future climate and land cover variables. We used this modelling framework to investigate how the impacts of changes to climate and land cover influenced biodiversity across ecoregions and elevation bands. There were large-scale climate- and land cover-driven changes in plant biodiversity across Madagascar, including both losses and gains in diversity. The sharpest declines in biodiversity were projected for the eastern escarpment and high elevation ecosystems. Sharp declines in diversity were driven by the combined climate-land cover scenarios; however, there were subtle, region-specific differences in model outputs for each scenario, where certain regions experienced relatively higher species loss under climate or land cover only models. We strongly caution that predicted future gains in plant diversity will depend on the development and maintenance of dispersal pathways that connect current and future suitable habitats. The forecast for Madagascar’s plant diversity in the face of future environmental change is worrying: regional diversity will continue to decrease in response to the combined effects of climate and land cover change, with habitats such as ericoid thickets and eastern lowland and sub-humid forests particularly vulnerable into the future.

## Introduction

Tropical biodiversity is being modified as a consequence of global environmental change [1–5]. In particular, climate and land cover change are driving a major reorganization of terrestrial biotic communities, each playing critical roles in determining future species compositions [6–8]. Whether the individual or combined pressures of these drivers will more negatively impact future biodiversity in the tropics is a complex issue. Climate change modulates species-specific responses to abiotic variables which affect species’ ranges, recruitment and survival [1]; meanwhile, land cover change affects local and regional species pools through disruption of species’ dispersal ability [9,10], driving local extinctions but also promoting colonization (e.g., for ‘matrix’ tolerant species). This limits the migration of intact plant and animal assemblages and contributes to major shifts in biodiversity for certain ecological communities [2,11].

In environments affected by climate change, flora and fauna must adapt to new temperature and precipitation extremes, where individuals may be pushed to the limits of their environmental tolerances [12]. Their survival depends on the rate at which they can migrate to different elevations, latitudes or regions otherwise suitable in terms of environmental conditions [13]. Whilst some species may adapt in response to climate change, those with longer generation times may not have sufficient time for natural selection to act [14]. As a result, climate change drives shifts in species’ geographic distributions [15], such that species tend to track their climatic niche—for instance toward higher elevations and latitudes when adapting to rising temperature [16,17] or downhill to optimise water balance [3]. Species confined to dry or drought-stressed regions may also benefit from increased water use efficiency, an advantage conferred on plants as a consequence of rising atmospheric CO_2_ concentrations [18].

Deforestation causes changes in albedo, evapotranspiration rates and water balance [19], establishing land-climate feedbacks that affect atmospheric circulation and rainfall [20]. Moreover, atmospheric changes associated with large-scale deforestation are not restricted to deforested habitats, but also influence climate over very large areas [21]. Whilst climate change acts at broad scales, land cover change may concentrate the effects of climate at the local scale [22]—a synergy that could result in a cycle whereby the intensified impacts of climate result in a higher rate of land cover change locally. Because of this, it is commonly assumed that the combined pressures from these drivers should lead to greater biodiversity loss than if their impacts were uncoupled [20]. However, the evidence to support these assertions remains sparse, as future predictions of how climate and land cover change affect species distributions and therefore biodiversity are difficult to model (although see [7] and [8]).

**Fig 1:**
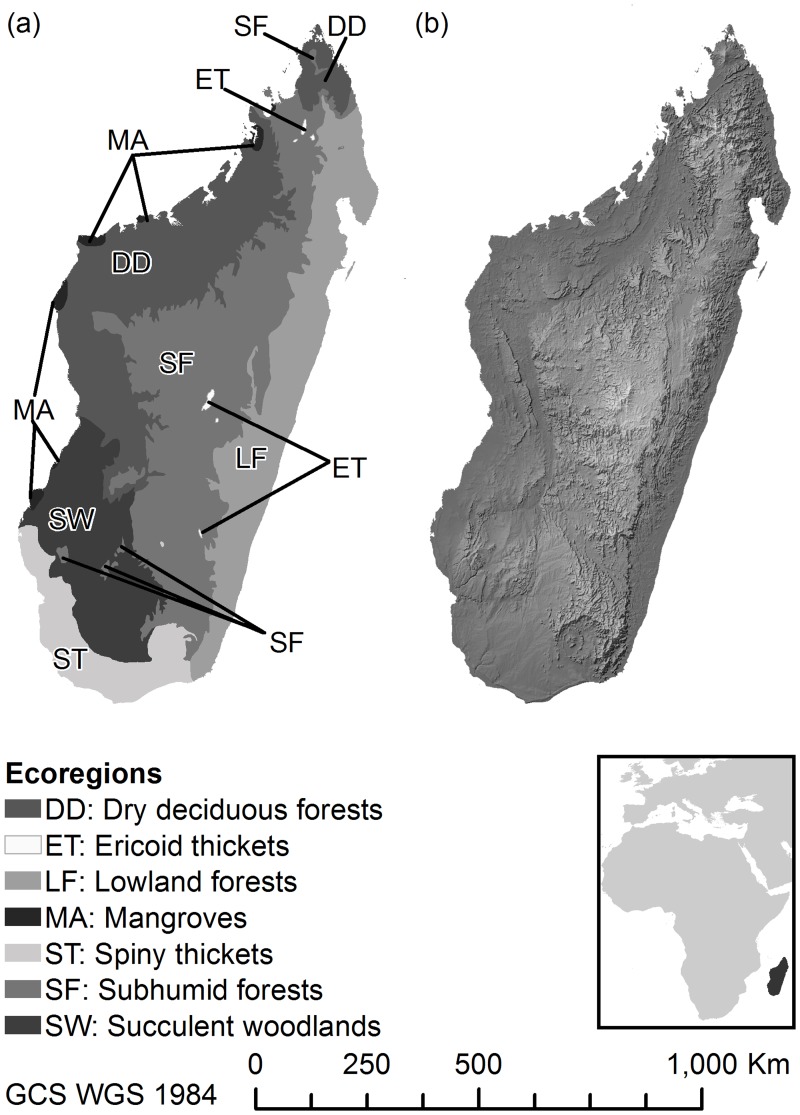
Site map showing the (a) ecoregions on which the analyses focused (excluding Mangroves) and (b) relief map of Madagascar, constructed using hill shade. Geographical Coordinate System (GCS) using the WGS1984 datum. Color figures available as S1 Fig.

Despite the ambiguities about the magnitude of change associated with future climate variables and land cover, tropical biodiversity is likely to be negatively affected [23]. This is because tropical species are restricted to a narrower range of climate variation [24], are often poor dispersers [25] and are already adapted to the warmest and wettest parts of the climate spectrum [26]. Therefore, the likelihood of them adapting further and rapidly migrating to new, suitable habitats may be limited—assuming that they have already been exposed to the most extreme climate variables in their current range. Furthermore, it has been hypothesized that there are biogeographical constraints on plant species in the tropics adapting to climate change by latitudinal shifts in distribution [27,28]. Plants are more likely to respond to climate warming through upslope range shifts [29], suggesting that high-elevation species in the tropics are more vulnerable to local extinction from climate change. However, they are less likely to be threatened by land cover change, as high elevation forests experience less deforestation and degradation than easily accessible lowland habitats. These observations suggest that the individual and combined impacts of climate and land cover change will have important but potentially varied implications for future plant biodiversity in the tropics. In this paper, we developed species distribution models (SDMs) calibrated for recent climate and land cover variables and projected to mid-century climates and predicted vegetation changes, to examine shifts in patterns of plant biodiversity in Madagascar (Fig 1). We focused on diversity patterns mediated by three scenarios: exclusively climate or land cover change and one that combines both. Our ultimate goal was to construct species richness maps that reflected the potential impact of these factors on biodiversity patterns in this mega-diversity hotspot.

We had two primary objectives for this study. First, we evaluated how the exclusive and combined effects of climate and land cover change influenced patterns of plant biodiversity (species richness) across Madagascar. Considering that our analyses were focused on country-wide patterns, we expected the scenario that incorporates both drivers of environmental change to have the largest negative impact on richness. Second, using the modelling framework established above, we quantified the magnitude of change in richness at different elevation ranges, as well as within major ecoregions across Madagascar. We expected the greatest decrease in richness to occur at the highest elevations under all scenarios, with the combined land–climate scenario exhibiting the largest decrease and the relative influence of the land cover only scenario showing the lowest decrease in diversity. We expected those ecoregions predicted to experience extreme shifts in climate (e.g., increasing temperatures in the south) or land cover (high rates of deforestation) to have noticeable changes in richness.

## Methods

### Study site

Madagascar forms part of one of 34 global biodiversity hotspots [30], as it possesses high levels of plant endemism [31], but also high rates of deforestation and degradation [32]. The high rate of endemism (plant and other taxa) in Madagascar can be explained not only by the island’s isolation from major landmasses, but also by its diverse and variable microclimates, as well as the contrast between the humid eastern escarpment and dry western biomes [33]. The climate associated with these regions (and throughout the island) is expected to change in coming years; projections of future temperature changes in Madagascar indicate increases of 1.1–2.6°C for the period 2046–2065, with the greatest level of warming occurring in the south and least in the north [34]. Precipitation will increase throughout the island in summer and becoming drier in winter along the southeast coast, but wetter in all other regions [34]. Just as climate is expected to vary, major changes in land cover have already been documented [35,36] and are expected to continue into the future [37]. For example, Harper *et al*. [34] report average deforestation rates in Madagascar from 1990–2000 at 0.9% yr—^1^, with humid and dry forests showing the greatest loss from *c*. 1950—*c*. 2000 of 43% and 41%, respectively. If forest fragmentation is taken into account, the consequences of land use change are even more dramatic. Relative to 1950s, in *c*. 2000 >45% of all forest occurred in patches (with a quarter in isolated patches <10 km^2^), and >80% within 1 km of a non-forest edge [35]. The majority of the endemic flora is forest dependent and areas outside forests typically harbour little biodiversity, as there is a sharp ecotone between forests and non-forests. Moreover, the biodiversity housed in the many habitats in Madagascar are vital for ecosystem services, directly influencing the livelihoods of millions of people in forest-dependent rural communities [38].

### Presence locations, climate and simulated land cover change scenarios

Species occurrence records and associated metadata (collection date and location) for vascular plant species within Madagascar were downloaded from Global Biodiversity Information Facility (GBIF: http://www.gbif.org/) and processed to remove duplicate records (defined as occurrences falling at the same coordinates at 1 km^2^). The data were subjected to further filters where we retained records that: (i) included greater than 10 occurrences; (ii) occurred over Madagascar’s landscapes rather than seascapes; (iii) were collected post-1980; and (iv) were less than 2876 meters above sea level (m.a.s.l). Other sampling biases, such as presence of synonyms and plant misidentification were not assessed and may inflate measures of species richness. After processing, there were 107630 genera and 69808 species occurrences, representing 828 distinct genera and 2186 species. On average, there were 118 occurrences per genus (median = 44 and SD = 201.7) and 27 occurrences per species (median = 19 and SD = 24.3). The climate variables were taken from WorldClim [39] recent (2000) and future scenarios, which were collected from a 30 arc-second resolution grid (i.e., 1 km^2^ resolution). Future scenarios represent a doubling of CO_2_, downscaled using the CCM2 model [40]. Doubling of CO_2_ represent a possible radiative forcing of 2.8Wm^-2^, with a probable temperature change of 2.7°C [41]. The climate variables included in the SDM were mean annual temperature (°C), minimum temperature (°C), maximum temperature (°C), mean annual precipitation (mm), minimum precipitation (mm), maximum precipitation (mm), annual water balance (mm), number of months with positive water balance (value between 1–12) and evapotranspiration rate (mm).

We used published sources of deforestation rates to estimate future changes in percentage land cover. We assumed a constant rate of land cover change across Madagascar, as it was not possible to reliably distinguish between different forest types at the scale of our analysis. Additionally, estimates of land cover change across Madagascar vary widely, making it challenging to arrive at reliable criteria by which to bound future rates of change [42]. In addition to Harper *et al*. (2007) [34] projections reported above, Allnutt *et al*. (2013) [43] report 40.4% forest loss for all of Madagascar between 1950–2000; Elmqvist *et al*. (2007) [44] estimate 7% decrease in total forest cover for dry forests between 1984–2000; and Grinand et al. (2013) [45] suggests deforestation rates of 0.93–2.33% yr^-1^ for humid forests and 0.46–1.117% yr^-1^ for the dry forest. Given this variation in estimates of land cover change, we opted for a low and conservative estimate of change based on ~30% of the reported deforestation rate from Harper *et al*. (2007) [34]. Therefore, we assumed that herb and forest cover would decrease at a rate of 0.28% yr^-1^, giving a decrease of 22% from the 2001 baseline to 2080.

This estimate of land cover change was used to simulate future deforestation / reforestation on a pixel-by-pixel basis within Madagascar through the year 2080 (i.e., each pixel was modelled as a separate entity, with no interaction with neighboring pixels). We simulated future land cover change using the land cover change scenario module of the Co$tingNature tool from PolicySupport.org [46]. This uses the MODIS VCF 2000 product to provide baseline fractional bare ground coverage [47] and then combines this with the newer 2010 fractional tree cover ([46], http://www.policysupport.org/simterra). The 2010 fractional herb cover is calculated such that,

$$H=100\;-\;(T_{2010}+B_{2000}),$$

where *H* represented the fractional herb cover, *T*_*2010*_ fractional tree cover [48], and *B*_*2000*_ fractional bare ground cover [47]. In this approach, Co$tingNature assumes that bare ground (e.g., rocky outcrops) are unlikely to change over time and tree cover loss is thus converted to herbaceous cover, which represents agriculture. The deforestation rate outlined above was applied on a pixel-by-pixel basis to the fractional tree cover, with the fractional cover of herbs increasing by an equivalent amount to the tree loss.

This represented a consistent level of forest degradation—from trees to herbs—across Madagascar, rather than pockets of intense deforestation. Whilst the latter may be a more realistic scenario, the simple deforestation model used here provides a suitable approach for exploring the combined effects of climate and land cover change on multiple species distributions at a relatively coarse scale (~1km), given the lack of more spatially explicit deforestation / degradation rates and scenarios available at the time. Therefore, Co$tingNature allowed us to develop environmental layers for recent and future tree and herbaceous coverage that were used in the Maxent SDMs. A bare soil layer was also produced, however, we did not include it in the SDMs.

These climate and land cover data layers were used to create three scenarios: (a) climate only; (b) land cover only; and (c) a combined model that coupled climate and land cover change. The recent values for these three scenarios were used to train Maxent models and their future values used to project SDMs into the future. That is to say, in order to assess change in richness, each scenario was developed with recent and future conditions and an SDM was generated under both conditions (e.g., recent climate only SDM for species X and future climate only SDM for species X).

### Modelling algorithm

SDMs were developed for each of the scenarios with Maxent software (Maxent v3.3.3k; [49]). Maxent has been shown to perform well in comparison to other approaches [50,51] and is known to perform well with small numbers of presence locations [52,53]. It relies on presence-only records to estimate the probability of occurrence for a species, which can then be used to discriminate suitable versus unsuitable areas. We implemented Maxent using the command line function for batch processing. Species With Data (SWD) file format, which includes presence records and environmental variables used for the input file.

### Model evaluation

We used area under the receiver operating characteristic curve (AUC)—a threshold independent index—to model predictive performance. Five-fold cross-validation was used and model performance was assessed on the held-out (i.e., test) folds. Cross-validation is a widely used resampling technique and has performed well in similar modelling studies [53]. AUC measures the ability of a model to discriminate between sites where a species is present and those where it is absent, which indicates the efficacy of the model for prioritizing areas in terms of their relative importance as habitat for a species. The AUC ranges from 0 to 1, where 1 indicates perfect discrimination and 0.5 suggests predictive discrimination is no better than random and values below 0.5 implies performance worse than random. We also used two threshold dependent indices—COR and Cohen’s Kappa (Kappa)—to assess model performance. COR is the point biserial correlation coefficient [50] and measures the ability of model predictions to discriminate between observed presence and absence [54]. Kappa measures whether the agreement between observed and predicted outcome is higher than that expected by chance.

### Decision thresholds

Constructing genus and species richness maps requires binary predictions (presence / absence) that establish a threshold value above which model output is considered to be a prediction of presence and below which a prediction of absence. The continuous distribution output in Maxent was converted to binary predictions using one of three threshold methods. The first criterion determined the threshold where positive observations were just as likely to be wrong as negative observations (i.e., point where “sensitivity” = “specificity”). Next, we used the criterion that minimized the mean of the error rate for correct observations and the error rate for incorrect observations (i.e., Youden’s Index). Lastly, we determined the threshold where the predicted prevalence was equal to the observed prevalence, where prevalence is the proportion of species occurrences among all sites (i.e., the sample frequency) [55]. Threshold values for the distribution maps were determined using all three techniques, thus producing binary maps that showed the probable distribution for each species and genus under each of the three threshold methods. These binary maps were then summed for each of the threshold techniques under each of the scenarios, resulting in 36 richness maps: three scenarios and three thresholds under recent and future conditions for genus and species (Table 1 and S1 Table).

**Table 1:** Model runs used to assess the potential impact of climate and land cover change on species and genera richness in Madagascar.

Threshold technique	Scenario	Taxonomic level	Mean Model performance (SD)			Pixel level loss/gain in richness		
			AUC	COR	Kappa	Minimum	Mean	Maximum
T1	Land cover only	Species	0.876 (0.05)	0.684 (0.08)	0.646 (0.12)	-699	-38.82	213
T3	Land cover only	Genera	0.859 (0.04)	0.638 (0.06)	0.599 (0.09)	-368	1.10	151
T3	Climate only	Species	0.923 (0.04)	0.765 (0.06)	0.731 (0.10)	-705	-39.50	199
T2	Climate only	Genera	0.907 (0.03)	0.721 (0.05)	0.681 (0.08)	-260	19.82	187
T1	Combined	Species	0.944 (0.03)	0.803 (0.06)	0.789 (0.10)	-791	-27.69	212
T2	Combined	Genera	0.927 (0.03)	0.758 (0.05)	0.735 (0.07)	-383	18.80	218

Only those thresholds selected for further analyses are shown (See S1 Table for complete table and discussion of model sensitivity to threshold selection). T1 runs were thresholded using the “sensitivity = specificity” rule; T2 runs were thresholded using Youden’s index; and T3 runs were thresholded using the “predicted prevalence = observed prevalence” rule. The mean values (and their associated standard deviations) for AUC, COR and Kappa are shown. Values for the model performance metrics are based on the average of 828 genera and 2186 species. The mean, minimum and maximum pixel level loss/gain in richness is also given.

Country-wide changes in richness for each scenario, under each threshold and for both taxonomic levels were calculated by subtracting the recent richness from the corresponding future richness, indicating genus/species richness on a per pixel basis. In order to present the most conservative estimates of the impact of environmental change, the model runs resulting in a mean change in overall richness closest to 0 were selected for further analysis (see S1 Table for full suite of thresholds).

### Changes in richness at regional-scale: elevation bands and ecoregions

In order to determine how the impacts of climate and land cover change vary across ecoregions and by elevation, a frequency distribution of changes in species and genera richness at the pixel level was constructed. We focused on seven ecoregions (Fig 1): (i) *dry deciduous forests*—dry tropical forests and woodlands occurring below 800 m in western region with a pronounced dry season; (ii) *lowland forests*—humid and moist tropical forest extending from low- to-mid-elevations in the eastern region; (iii) *sub-humid forests*—consists of a diversity of habitats and corresponds broadly to mid-elevation forests in central and southern regions, as well as northern highlands and upland habitats; (iv) *succulent woodlands*—occurs in the southwestern region and is comprised of deciduous woodland and spiny and succulent thicket; (v) *spiny thickets*—occurs in south and southwest consisting of low succulent and spiny thicket dominated by Euphorbiaceae and locally endemic Didiereaceae; (vi) *ericoid thickets*—occurs on the upper slopes of the four major mountain massifs and consists of a large number of endemics in Ericaceae, Asteraceae and Podocarpaceae; and (vii) *mangroves*—occurs mostly along most of the western coast [56]. Mangroves are presented in the site map and species/genera maps for completeness. They are not discussed in the results, because they are not speciose communities (i.e., ca. 10 species) and face very different pressures and impacts than other ecoregions.

Two methods were used to define elevation bands: one created bands of equal elevation intervals (but with varying areal extent) and the second created bands of equal area (but with varying elevation intervals). Seven equal elevation interval bands were created at 390 m intervals (hereafter equal interval bands). The highest band using this method contained just 0.02% of Madagascar’s areal extent, whilst the lowest band contained 48%. In order to account for this variation in areal extent between bands, genus and species richness calculated within equal interval bands were normalized by the area of that band. The second method involved creating seven bands of equal area, with each covering approximately 105 000 km^2^ (hereafter equal area bands). The highest elevation band using this method account for 61% of Madagascar’s total elevation range (1067–2744 m), whereas the lowest elevation band accounted for 0.02% (0–62 m). The value of the DEM pixel with the greatest elevation was 2744 m; whilst Maromokotra in Tsaratanana is 2876 m, the pixels of the DEM are ~1km and individual peaks are not accounted for.

Using the zonal statistics tool in ArcMap v 10.1 [57], the change in mean richness (and associated standard deviation) within each of the zones under each of the scenarios was calculated. The results are presented as absolute changes in richness (i.e., gain / loss in species and genera within the zone of interest), thus allowing interpretation of the number of genera and species likely to be lost overall. Where necessary, gains / losses in species and genera richness were normalized by the pixel count of the zone of interest, allowing comparison between zones of different sizes. This is particularly important when considering changes along an elevation gradient with equal vertical intervals, as the zones at the highest elevations are likely to be significantly smaller. We refrain from making species-specific predictions, as our analyses are too coarse-grained to detect distinct changes in geographic ranges.

The data were analysed using Kingston University High Performance Computing facility, which offered sixteen dedicated servers, multiple, simultaneously running simulations and scripted automatic execution. This facilitated processing and storage of 828 genera and 2186 species for six scenarios and 18 different thresholds. We used Maxent’s batch file feature, without the interactive GUI overhead, to develop species distribution maps; outputs for which were exported to R-statistical software [58] for determining threshold values and model performance using PresenceAbsence package [59]. The interface between Maxent, R and the parallel processing servers was managed with a separate Unix script. The species richness maps (developed from the threshold output files) were assembled in ArcGIS using PCRaster python (http://pcraster.geo.uu.nl) with all subsequent analysis implemented in ArcGIS v 10.1 [57].

## Results

Four primary patterns in Madagascan plant diversity were evident: (i) there was a large-scale heterogeneous pattern of diversity change, where some regions showed sharp declines and others increases; (ii) there were region-specific differences in the impact of each driver on biodiversity, where certain regions were influenced more by either climate or land cover, rather than the combined effects of both; (iii) the sharpest declines in biodiversity were projected for the eastern escarpment and ericoid thickets; and (iv) diversity at the highest elevations were projected to experience sharp declines. Model performance, as measured by mean AUC, gave excellent to outstanding discrimination [60] while mean Kappa exhibited good to excellent results, ranging from 0.599–0.789 [61] and mean COR ranged from 0.638–0.803 (Table 1).

### Changes in biodiversity: Regional-scale

**Fig 2:**
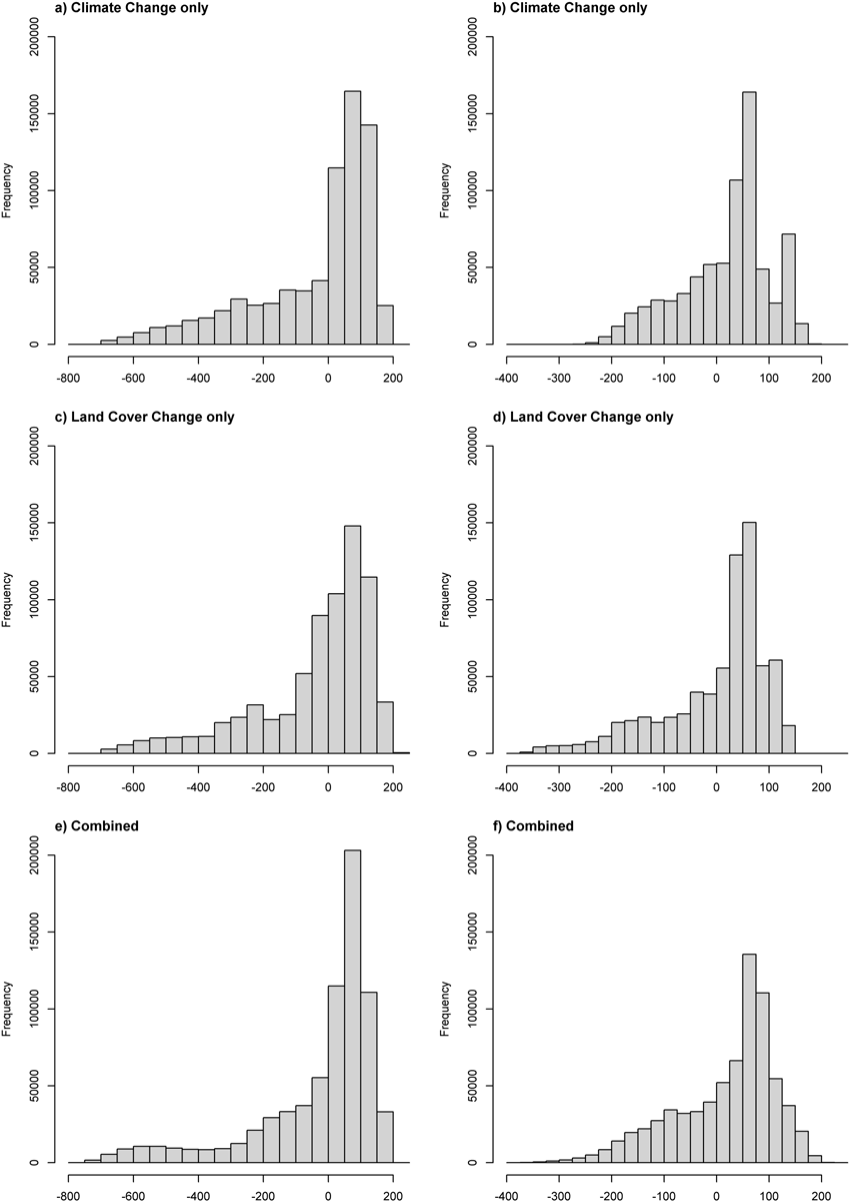
Histogram showing pixel-level gains and losses for species (a, c and e) and genera (b, d and f) for the three scenarios, climate only, land cover only and combined.

**Fig 3:**
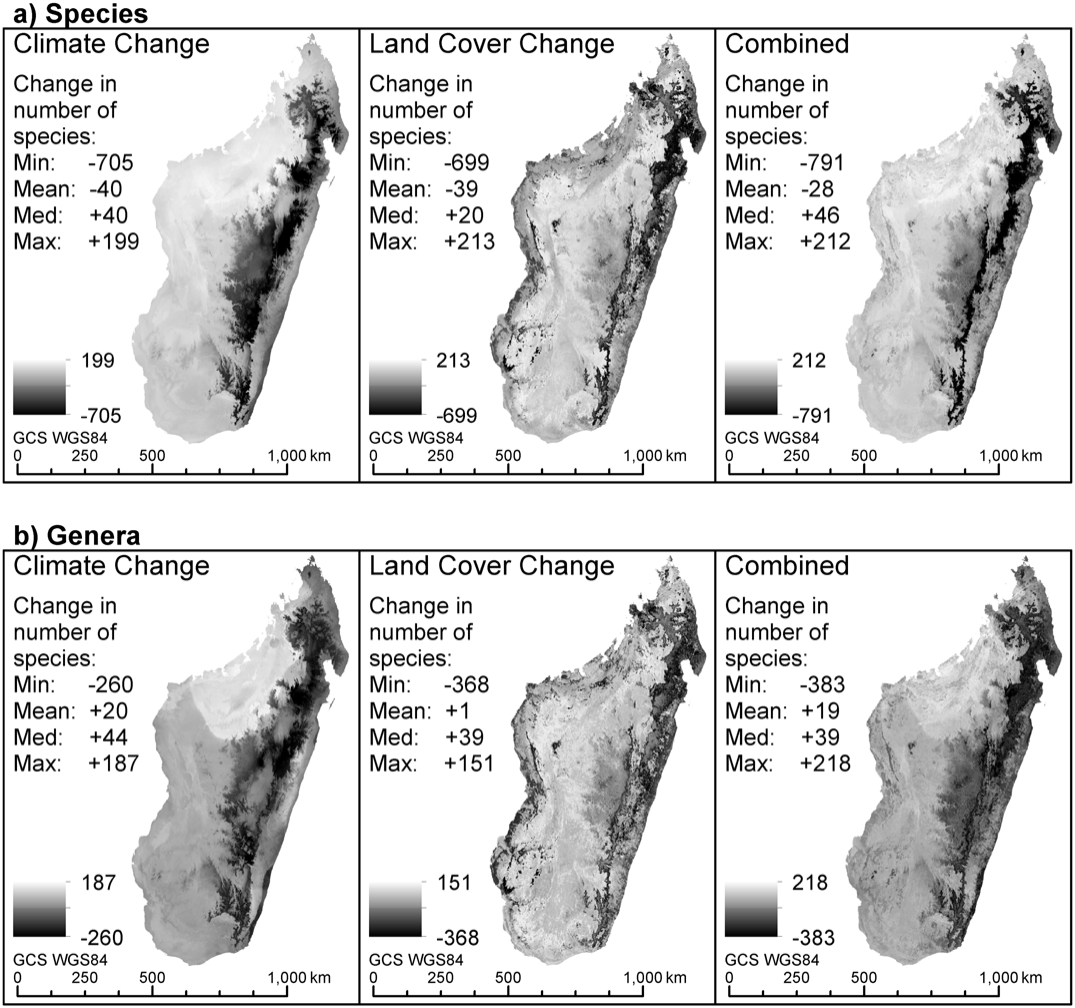
Change in richness from recent (2000) conditions for climate only, land cover only and combined scenarios for (a) species and (b) genera. Darker areas indicate species and genera richness losses and lighter areas indicate gains. Geographical Coordinate System (GCS) using the WGS1984 datum. Color figures available as S2 Fig and S3 Fig.

Model projections suggested that there would be large-scale climate- and land-cover-driven shifts in plant distributions across Madagascar, where species/genera gains were predicted for certain regions and losses for others. The three scenarios show similar overall frequency distributions of taxonomic loss. The skew in distribution indicates that most locations will gain some species/genera; however, the long left-hand tail—indicating loss of species—suggests that other locations will lose large numbers of species/genera, as well (Fig 2). Moreover, the combined climate-land cover scenario did not always have the strongest negative effects on richness, suggesting that the influence of each driver was not concentrated when acting together (Fig 2). That is to say, the left-skewness of the histograms for the combined scenario was not predicted to double, nor increase at a rate that suggested an additive or synergistic effect of climate and land cover (Fig 2; Fig 3a and 3b).

Predicted changes in plant richness at the country-wide scale were equivocal. If assessed using median values, species richness was predicted to increase under all three scenarios (Fig 3a; median increase of 40, 20 and 46 for climate only, land cover only and combined respectively). On the other hand, the mean values for species richness showed small decreases (Fig 3a; mean decrease of 40, 39 and 28 for climate only, land cover only and combined respectively). Genera richness was predicted to increase under both measures of central tendency for all three scenarios (Fig 3b). Plant species richness declined sharply in a few spatially concentrated areas, with the land cover and combined scenarios driving relatively larger losses than climate only (Fig 3a and 3b).

The sharpest declines in floristic richness occurred along the eastern escarpment and although consistent across the three scenarios, were driven mostly by changes in climate. Land cover seemed to drive much of the loss in the western regions (Fig 3a). Moderate increases in species richness were predicted in the northwest under both the climate only and combined scenarios, whilst this pattern was less apparent in the land cover only scenario (Fig 3a). Changes in genus richness follow the same spatial patterns as those for species richness, although the gains in the northwest under the climate only scenario were more pronounced and this is also reflected in the combined scenario (Fig 3b). All three scenarios suggest that areas in the northwest and less so in the south-southwest will experience an increase in generic richness, with country-wide median increases of 44, 39 and 39 genera for the climate, land-cover and combined scenarios respectively (Fig 3b).

### Changes in biodiversity: elevation ranges and ecoregions

**Fig 4:**
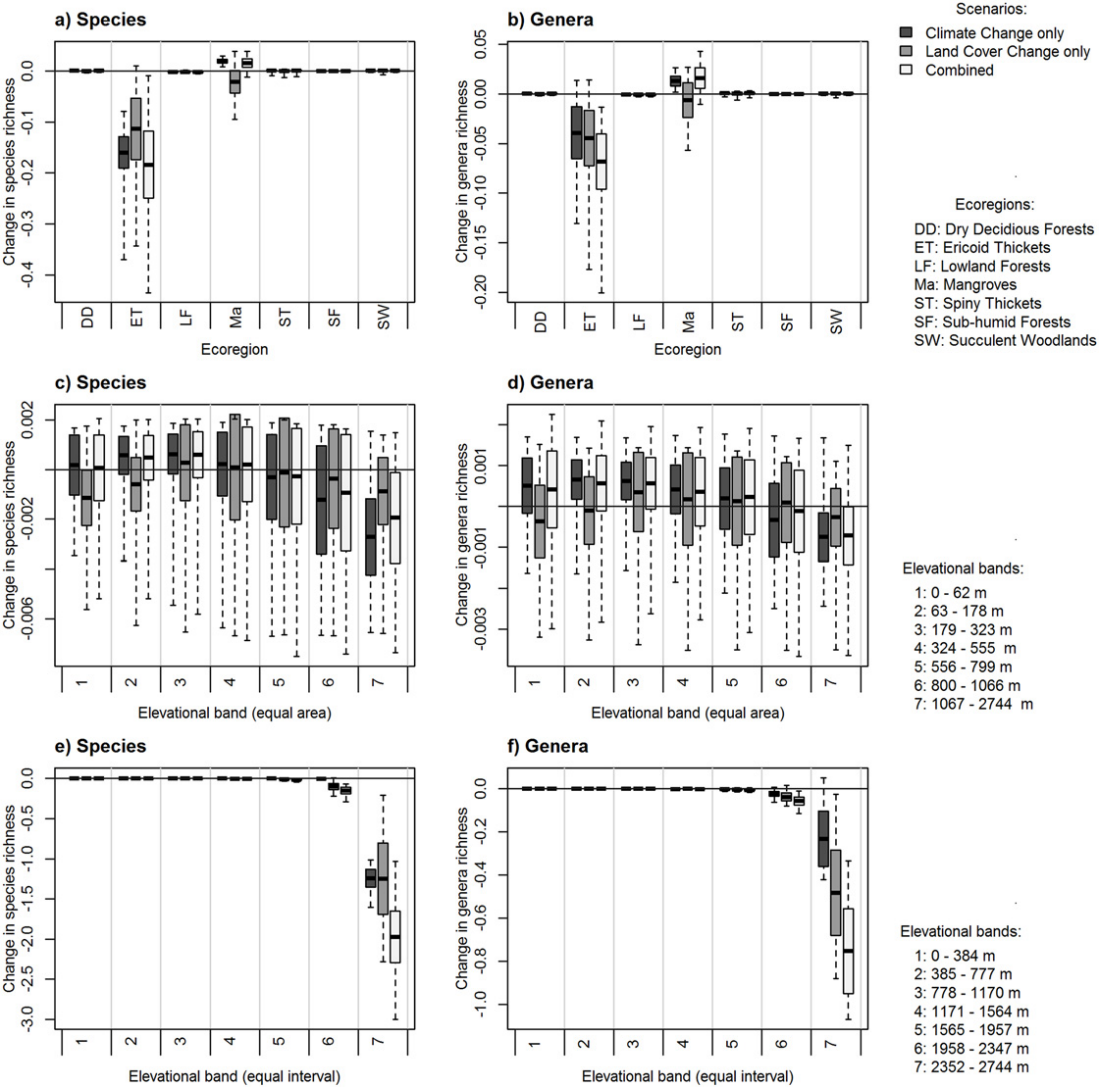
Loss and gain in plant species and genera richness (measured on a pixel-by-pixel basis and normalized by area, km^2^) across different zonal systems for (a, b) ecoregions found in Madagascar, (c, d) equal area elevation bands (each with an area of approximately 105 000 km^2^), and (e, f) equal interval elevation bands (at intervals of 390 m). Changes under each scenario are shown as: climate only scenario (dark grey), land cover only scenario (mid-grey) and the combined scenario (light grey). The mean is indicated with a solid horizontal line, the shaded areas represent ±1 standard deviation, whilst dashed lines extend to the minimum and maximum values.

**Fig 5:**
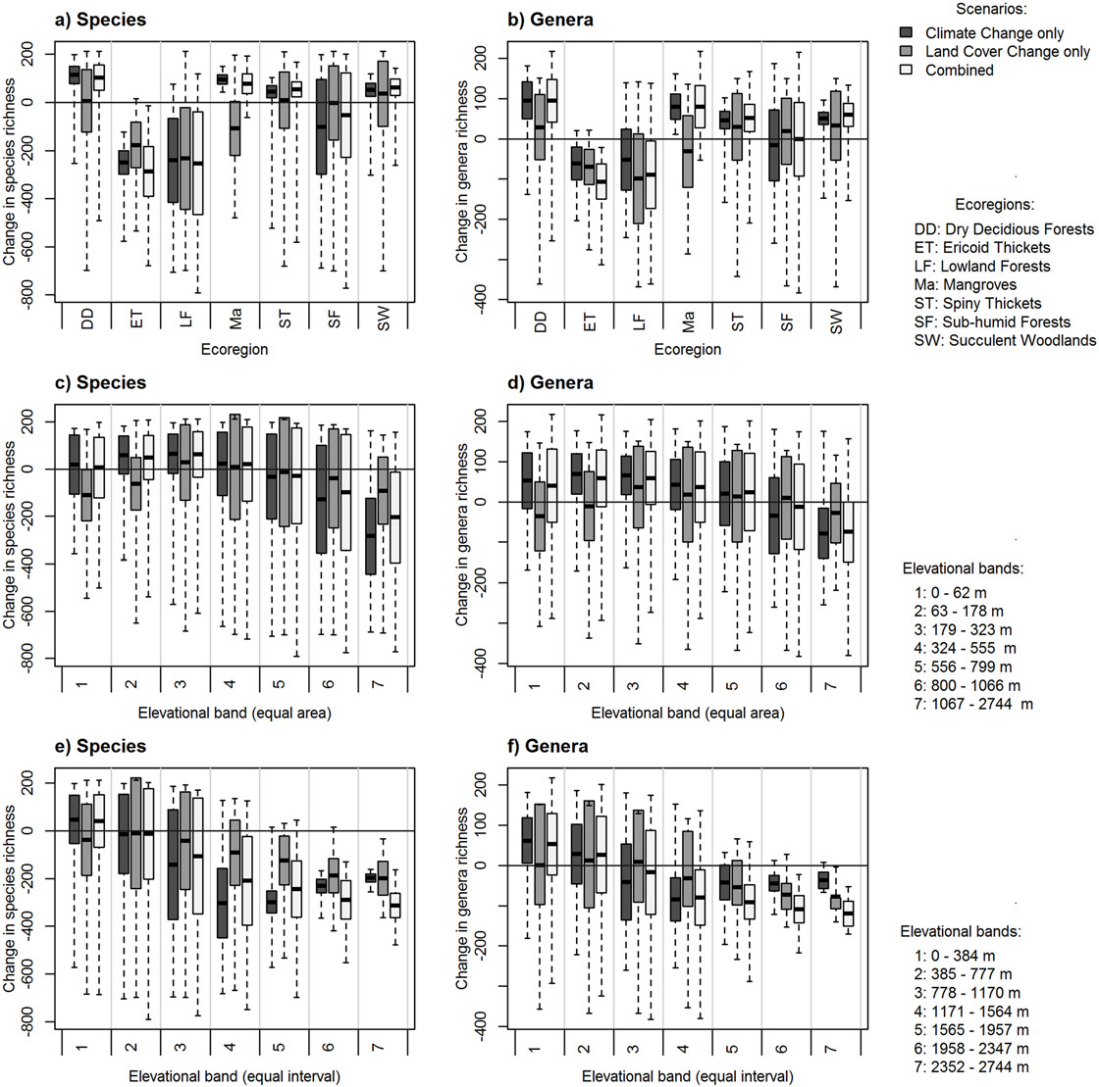
Changes in species and genera richness across different zonal systems, showing the absolute changes in richness for (a, b) ecoregions found in Madagascar, (c, d) equal area elevation bands (each with an area of approximately 105 000 km^2^, (e, f) equal interval elevation bands (at intervals of 390 m). The changes under each scenario are shown, with the climate change only scenario represented with the darkest grey, the land-cover change only scenario represented with the mid-grey and the combined scenario represented in the light grey. The mean is indicated with a solid horizontal line, the shaded areas represent ±1 standard deviation, whilst dashed lines extend to the minimum and maximum values.

We present both proportional (normalized by area) and absolute losses in genus/species richness within elevation zones and ecoregions. Focusing first on proportional losses—there was a slight decrease in richness using equal area elevation bands for both genera and species under all scenarios at the highest elevations (Fig 4). This decrease was highlighted for the equal interval elevation bands, where the largest losses of diversity were also projected for the highest elevations (Fig 4). That is to say, if we controlled for area, losses in species richness was greatest at the highest elevations under the combined scenario, with the land cover only projections showing the least amount of genus/species loss (Fig 4). However, this signal was less apparent when using the absolute values (Fig 5).

In terms of proportional losses in ecoregions, ericoid thickets and lowland forests were predicted to suffer the greatest losses of both species (losing on average approximately 200 species under each scenario) and genera (losing a mean of approximately 100 genera under each scenario; Fig 4), driven by the combined effects of climate and land cover. This pattern remained under projections based on absolute losses—ericoid thickets were still predicted to lose both species and genera (approximately 0.05 genera km^-2^ and 1.5 species km^-2^; Fig 5).

## Discussion

### Predicted changes in biodiversity at regional-scale

In this study, we used SDMs to explore whether future changes in climate and land cover will drive shifts in plant species and genera richness in Madagascar. Our results document large-scale climate- and land-cover-driven shifts in diversity across Madagascar, where species and genera declines were forecast for certain regions, while others were predicted to show gains. Notably, the largest decline in diversity was predicted for humid forests in the eastern escarpment and high-montane ericoid thickets. These habitats are two of the most important in the country, ericoid thickets for high rates of endemism and eastern tropical forests for having the highest plant diversity in the country [31]. The predicted decline in richness for forests along the eastern escarpment is consistent with observed and predicted changes to climate and land cover for the eastern region [62]. For example, recent observations, spanning from 1961–2005, show a reduction in rainfall and increase in the numbers of consecutive dry days, which supports Tadross (2008) [33] prediction that plants in the eastern region are expected to show increased levels of drought stress. Also, eastern escarpment forests are highly fragmented, have one of the highest deforestation rates in the country and are vulnerable to continued forest loss [45,63]. Plants in the high-montane ericoid thickets would also experience warming temperature trends [16] and they are locally restricted to high elevations (e.g., 1900 m—2050 m) of the four major mountain massifs on the island. Both factors would make species/genera in that ecoregion vulnerable to future declines in richness, which our models substantiate.

### Changes in biodiversity: elevation ranges and ecoregions

Although we expected the combined climate-land cover scenario to drive large declines in biodiversity, this was not always the case. This general finding runs contrary to our expectation, as well as results from other modeling studies that suggest the effects of climate and land cover might be concentrated when acting together [61]. With that said, however, the ericoid thickets once again stand out as particularly vulnerable. Our models predicted substantial declines in future vegetation richness in this ecoregion under all change scenarios, but particularly for the combined climate-land scenario. This might be due to the specialized nature of environments with high levels of endemism, since dispersal to suitable, future habitats will be less likely. Of equal importance, as Feeley and Rehm (2012) [61] suggest, this may be because species migration to more suitable environments in response to climate change may be hindered by deforestation, since the latter may increase dispersal distances needed to establish viable populations in future environments.

Several regions experienced relatively lower species loss under the combined scenario compared to climate or land cover alone. Lowland and sub-humid forests suffered the largest declines under the climate scenario, where the possible effects of climate change exceeded the effects of land cover. These results support findings of Feeley *et al*. (2012) [7], who suggests that climate variables—particularly increasing temperature—are the dominant drivers affecting future plant diversity in the Amazon. In Madagascar, this may lead to sharp declines in lowland tropical diversity over the next century unless organisms are able to tolerate or adapt to thermal extremes, in particular the predicted increase of 1.1–2.6°C over the next century [34]. The picture is different in the western part of the country (i.e., dry deciduous forest and succulent woodlands), where (relatively limited) species loss is driven mostly by land cover change rather than climate. These results suggest region-specific differences in the impact of each driver on biodiversity in Madagascar, which may be modulated more by either climate or land cover, rather than the combined effects of both. Similar patterns have been documented by other studies, with land cover dominating over climate change in western Amazonia [7], as well as having a bigger impact on biodiversity in Asia-Oceania than in Latin America and Africa [8].

When we controlled for area, there were no noticeable change in diversity at the lower elevation bands (i.e., 0 m— 1957m), but there was increasing loss of diversity between 1958 m—2347 m and considerable loss between 2352 m—2744 m. We envisage two explanations for this observation. First, species range contractions and extinctions are more likely to occur at higher elevations, since there is a physical constraint on elevation at mountaintops and plants can no longer track shifting climatic conditions [27]. In the tropics, species are more likely to respond to climate–driven temperature change by shifting their range by elevation through upslope migration, rather than latitude [29]. This is particularly true for high elevation endemics, such as plants found in ericoid thickets that occupy a narrow climate space [16,64]. Alternatively, a decrease in richness at high elevation may be linked to the inability of plants to migrate to suitable climatic conditions since current and future suitable habitats do not overlap (i.e., range-shift gaps *sensu* Colwell et al. (2008) [27], which is an acute problem for low–and–high–elevation plant species [17]. Our study does not address these questions, but they are areas of continuing inquiry. What seems most clear from our study is that high elevation plants will contract their geographic range in response to the combined effects of climate and land cover change. The likely consequence of either alternative explanation may be that Madagascar’s high elevation species, particularly endemics, will become increasingly threatened into the future [65].

Equally important, the northern and western regions of Madagascar—encompassing dry deciduous and sub-humid forests and spiny thickets—are predicted to show gains in diversity under our scenarios. This may be a consequence of the relatively small increase in temperature that is projected for that region [34], if, again, it is assumed that temperature will be the dominant driver affecting biodiversity in the tropics [66]. That assumption should be made with a degree of caution, since temperature and precipitation interact strongly and results based solely on temperature can be confounded by precipitation and seasonality [64,67]. This result is also strongly dependent on intact habitats providing dispersal corridors for new colonizations, an unlikely prospect given the high rate of deforestation associated with dry forests in Madagascar [45].

### Evaluation of results

It is necessary to highlight a few important caveats regarding our model-based findings. First, increased diversity due to climate change does not indicate ‘re-vegetation’, instead it implies colonization possibilities—regions that once exhibited unsuitable climate for certain plants may in the future be within the limits of their environmental tolerances. That is to say, plant diversity may theoretically increase in local areas (e.g., NW) due to climate change (and in spite of deforestation) because these areas become climatically hospitable for a larger number of species that are currently found only in other regions. However, our models do not consider any type of dispersal mechanism. Therefore, gains in diversity would only occur if colonization pathways were present, which is unrealistic given on-going deforestation surrounding natural forests in Madagascar [43,63]. Second, our analyses examined regional–scale changes in diversity without explicitly investigating species–specific range shifts. Therefore, losses in biodiversity are most likely driven by shifts in species’ distribution, leading to local extinction, rather than global extinction. Next, we do not explore the importance of feedback loops between climate and land cover change on future plant distributions. It has become increasingly apparent that the direct impacts of climate change on species’ distributions may be less important than the increase in habitat loss as a result of human adaptation to climate change [68]. Madagascar is a strong example of this—forests are threatened primarily by slash and burn agriculture (known as *tavy* in Madagascar), which is expected to increase in response to increasingly unpredictable rainfall [34], in turn reducing the productivity of existing agricultural systems and forcing local communities to cultivate a greater land area by cutting down more forests. Feedback loops are expected to change patterns of deforestation as rural subsistence communities adapt to changing climatic conditions. Consequently, potential dispersal corridors may be closely linked to the intensity of prospective feedback loops. Finally, the study examined large–scale patterns of plant diversity, without focusing on a certain class of vegetation (e.g., endemic or utilitarian species). Using richness as the key diversity metric might suggest that all species/genera are equal in ecological importance or conservation value, and we recognize that is not the case. However, contrasting regional–scale diversity patterns can be instructive and valuable for understanding how major drivers of biodiversity change may vary across ecoregions [69,70].

In conclusion, our study suggests considerable heterogeneity in future patterns of diversity in response to climate and land cover change. Despite some sharp declines in richness (e.g., eastern lowland forests), there were also some projected increases in plant diversity, for example the northern and western regions (e.g., dry deciduous and sub-humid forests). This latter result, however, depends on the unlikely presence of colonization pathways. Moreover, Madagascar’s plant diversity is vulnerable to both individual and combined effects of climate and land cover change, suggesting that depending on the region, priorities may be placed on mitigating the effects of both drivers or on each separately; even if that may be challenging from a practical standpoint. Our results also suggest that high elevation endemics are particularly vulnerable. Whether a subset of these species will be able to shift their distribution to suitable habitats will be determined by two essential factors—the probability of colonizing safe sites within their environmental tolerances and the presence of dispersal corridors. Considering the global importance of Madagascar as a conservation priority and its status as a tropical, developing country undergoing similar pressures to other high biodiversity nations, there is a continuing need to understand how climate and land cover change will affect plant diversity in that country.

## Supporting Information

S1 Fig Color site map. Site map showing the (a) seven ecoregions on which the analyses focused and (b) relief map of Madagascar, constructed using hill shade. Geographical Coordinate System (GCS) using the WGS1984 datum.
(TIF)

S2 Fig Color species change map. Color version depicting change in species richness from recent (2000) conditions for climate only, land cover only and combined scenarios for (a) species. Geographical Coordinate System (GCS) using the WGS1984 datum.
(TIF)

S3 Fig Color genera change map. Color version depicting change in genera richness from recent (2000) conditions for climate only, land cover only and combined scenarios. Geographical Coordinate System (GCS) using the WGS1984 datum.
(TIF)

S1 Table Complete Model runs used to assess the potential impact of climate and land use change on species and genera richness in Madagascar. Those runs selected for further analyses are indicated with *. T1 runs were thresholded using the “sensitivity = specificity” rule, T2 runs were thresholded using Youden’s index and T3 runs were thresholded using the “predicted prevalence = observed prevalence” rule.
(DOCX)
