# Supplementary figures and images for: Predicting Plant Diversity Patterns in Madagascar: Understanding the Effects of Climate and Land Cover Change in a Biodiversity Hotspot
Source: PLoS One. 2015 Apr 9;10(4):e0122721. doi: 10.1371/journal.pone.0122721 (PMC4391717; doi:10.1371/journal.pone.0122721)

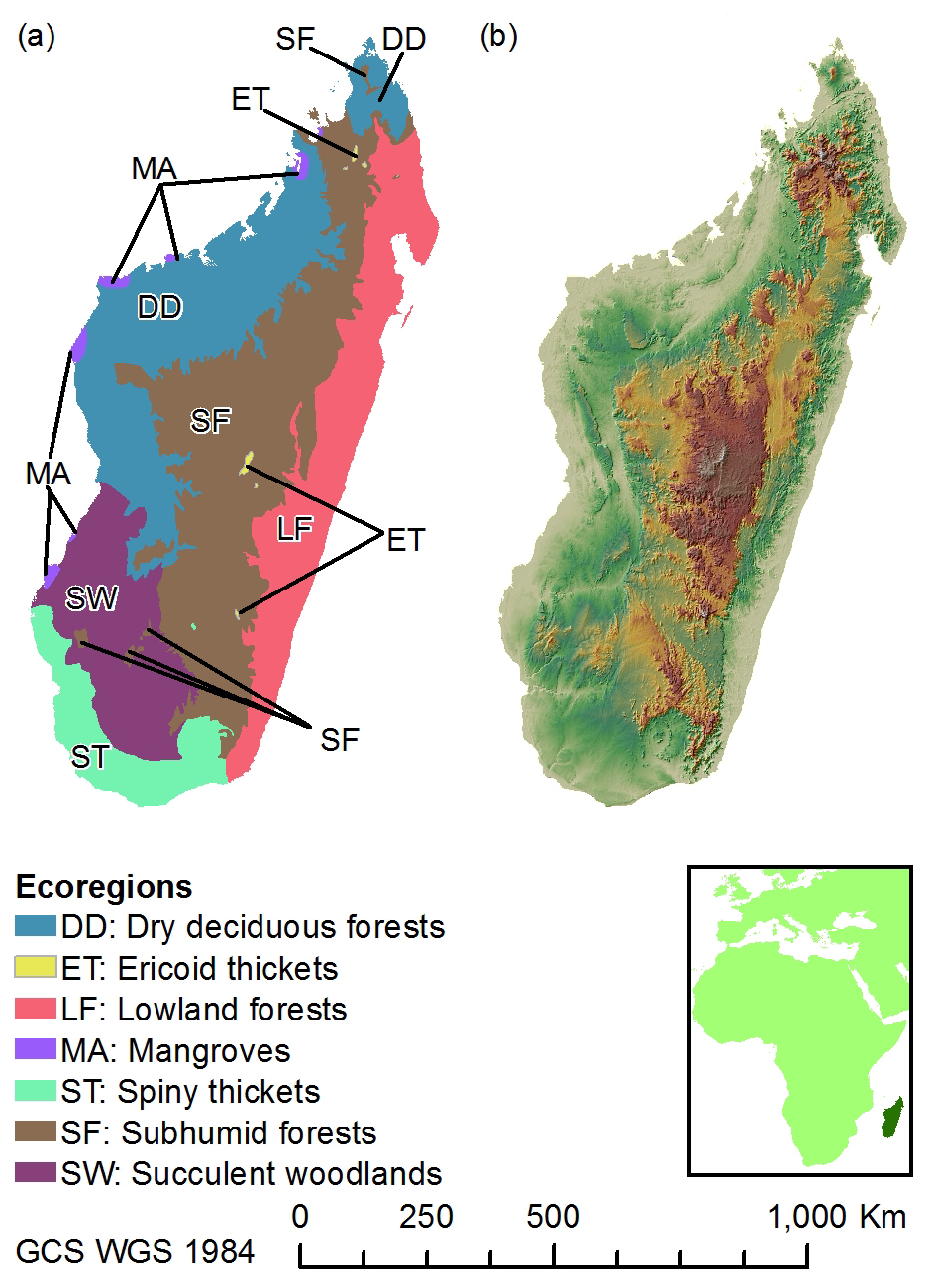

Supplement: S1 Fig — Site map showing the (a) seven ecoregions on which the analyses focused and (b) relief map of Madagascar, constructed using hill shade. Geographical Coordinate System (GCS) using the WGS1984 datum. (TIF) [file pone.0122721.s001.tif]

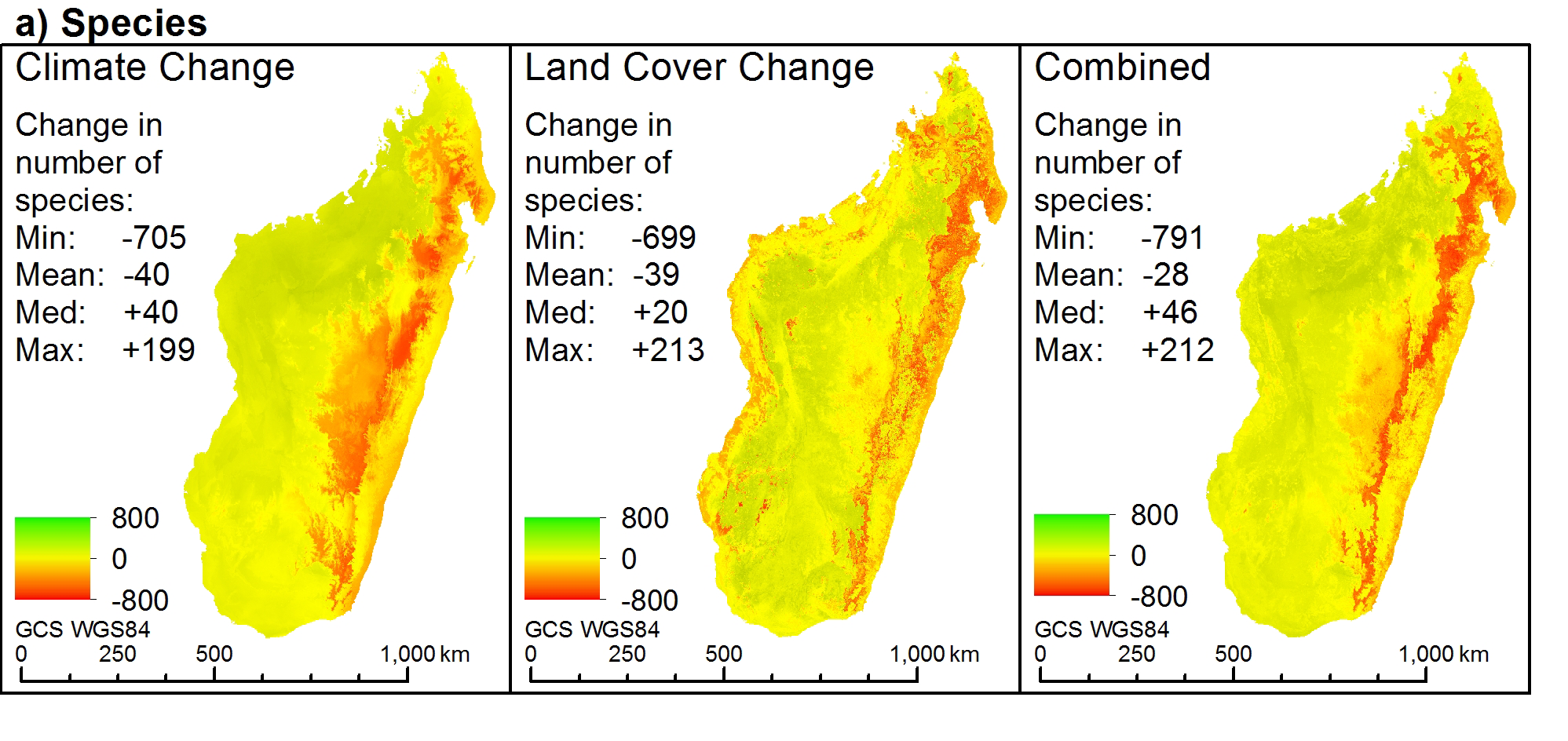

Supplement: S2 Fig — Color version depicting change in species richness from recent (2000) conditions for climate only, land cover only and combined scenarios for (a) species. Geographical Coordinate System (GCS) using the WGS1984 datum. (TIF) [file pone.0122721.s002.tif]

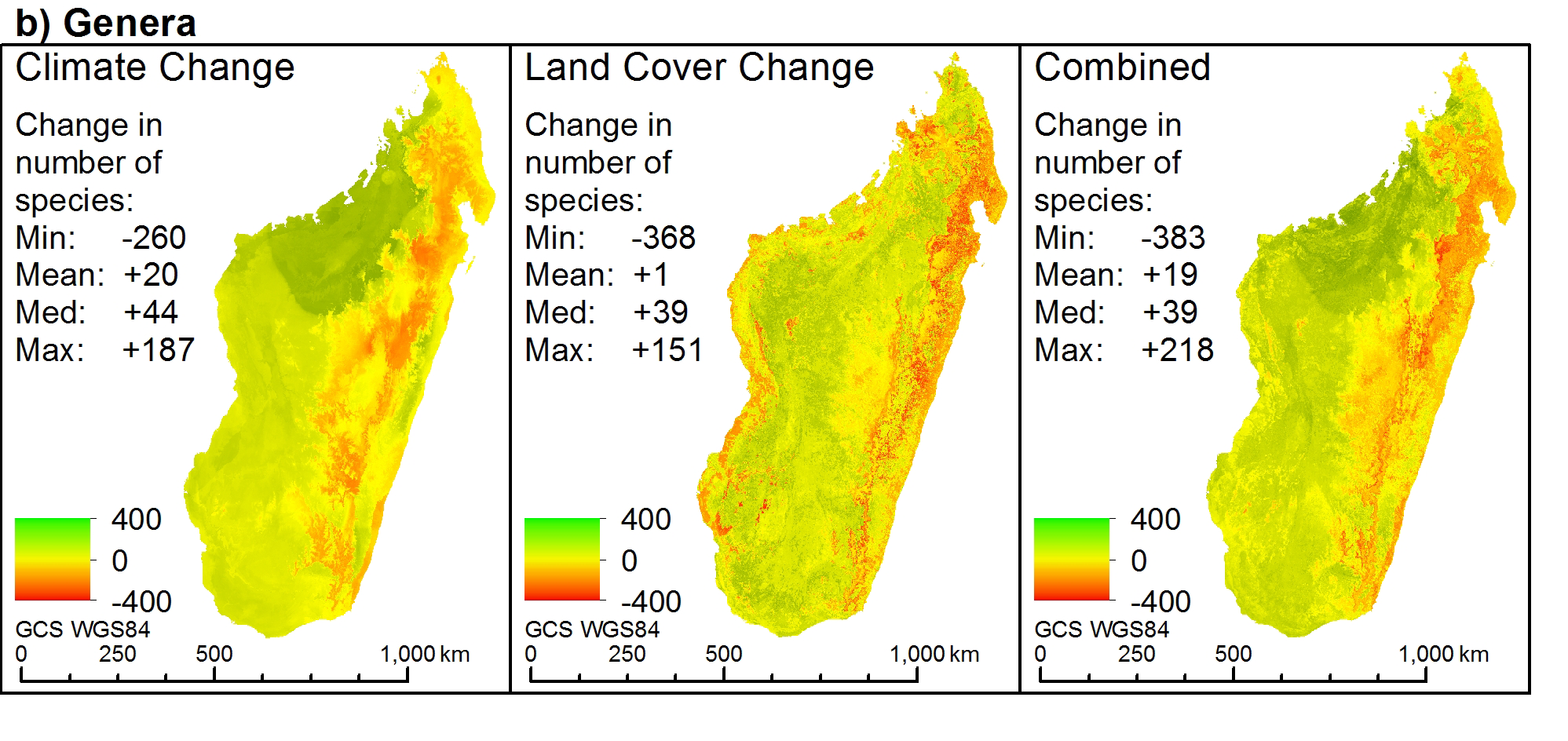

Supplement: S3 Fig — Color version depicting change in genera richness from recent (2000) conditions for climate only, land cover only and combined scenarios. Geographical Coordinate System (GCS) using the WGS1984 datum. (TIF) [file pone.0122721.s003.tif]
