# Supplementary material for: Predicting Plant Diversity Patterns in Madagascar: Understanding the Effects of Climate and Land Cover Change in a Biodiversity Hotspot
Source: PLoS One. 2015 Apr 9;10(4):e0122721. doi: 10.1371/journal.pone.0122721 (PMC4391717; doi:10.1371/journal.pone.0122721)
Supplement: S1 Table — Those runs selected for further analyses are indicated with *. T1 runs were thresholded using the “sensitivity = specificity” rule, T2 runs were thresholded using Youden’s index and T3 runs were thresholded using the “predicted prevalence = observed prevalence” rule. (DOCX) [file pone.0122721.s004.docx]

**Table S1.** Model runs used to assess the potential impact of climate and land use change on species and genera richness in Madagascar. Those runs selected for further analyses are indicated with *. T1 runs were thresholded using the “sensitivity = specificity” rule, T2 runs were thresholded using Youden’s index and T3 runs were thresholded using the “predicted prevalence = observed prevalence” rule.

| Threshold technique | Change Scenario | Taxonomic level | Pixel level loss/gain in richness | | |
| --- | --- | --- | --- | --- | --- |
|  |  |  | Minimum | Mean | Maximum |
| T1* | Land Use Only | Species | -699 | -38.82 | 213 |
| T2 | Land Use Only | Species | -734 | -57.88 | 186 |
| T3 | Land Use Only | Species | -702 | -38.85 | 215 |
| T1 | Land Use Only | Genus | -368 | 1.12 | 151 |
| T2 | Land Use Only | Genus | -397 | -4.63 | 165 |
| T3* | Land Use Only | Genus | -368 | 1.10 | 151 |
| T1 | Climate Only | Species | -703 | -39.54 | 201 |
| T2 | Climate Only | Species | -680 | -59.87 | 204 |
| T3* | Climate Only | Species | -705 | -39.50 | 199 |
| T1 | Climate Only | Genus | -257 | 23.67 | 184 |
| T2* | Climate Only | Genus | -260 | 19.82 | 187 |
| T3 | Climate Only | Genus | -257 | 23.67 | 183 |
| T1* | Combined | Species | -791 | -27.69 | 212 |
| T2 | Combined | Species | -753 | -41.69 | 224 |
| T3 | Combined | Species | -791 | -27.73 | 211 |
| T1 | Combined | Genus | -358 | 22.75 | 203 |
| T2* | Combined | Genus | -383 | 18.80 | 218 |
| T3 | Combined | Genus | -357 | 22.76 | 204 |

*Thresholding*

There was little consistency in the thresholding method that resulted in the lowest mean change in richness, with runs based on all three threshold techniques for all scenarios and taxonomic levels. For the land-cover only scenario, the “sensitivity = specificity” thresholding technique (T1) was selected for modeling species richness (predicting a mean loss of 39 species per pixel across Madagascar), whilst the “predicted prevalence = observed prevalence” technique (T3) was selected for genera richness (predicting a mean gain of 1 genera per pixel across Madagascar). Under the climate change only scenario, T3 was selected for modeling species richness, whilst the Youden’s index method (T2) was selected for genera richness (predicting a mean loss of 40 species per pixel and a mean gain of 20 genera per pixel respectively). For the combined change scenario, T1 was selected for modeling species richness (predicting a mean loss of 28 species per pixel) and T2 was selected for genera richness (predicting a mean gain of 19 genera per pixel).
